# Multiple Abdominal Desmoplastic Small Round-Cell Tumors Treated With Fan Beam Computed Tomography-Guided Adaptive Radiotherapy (FBCT-gART): A Case Report

**DOI:** 10.7759/cureus.69785

**Published:** 2024-09-20

**Authors:** Haohua Wang, Xiang Zhang, Jinbo Yue

**Affiliations:** 1 Radiation Oncology, Shandong First Medical University and Shandong Academy of Medical Sciences, Jinan, CHN

**Keywords:** desmoplastic small round cell tumor, ct-guided adaptive radiotherapy, fan beam ct, adaptive radiotherapy, radiotherapy

## Abstract

Desmoplastic small round cell tumor (DSRCT) is a rare and highly aggressive soft tissue tumor that predominantly affects the abdominal and pelvic regions of adolescent males. This case report presents our clinical experience of treating a 33-year-old male with multifocal peritoneal DSRCT using fan beam computed tomography-guided adaptive radiotherapy (FBCT-gART). The patient presented with abdominal pain and was diagnosed with DSRCT following imaging and biopsy. Despite initial treatment with surgery, chemotherapy, and targeted therapy, the patient experienced multifocal peritoneal recurrence. Due to the considerable mobility of the abdominal tumors and the associated risks to adjacent critical organs, the patient underwent daily online FBCT-gART. The prescribed dose regimen was 54 Gy delivered in 27 fractions at 2 Gy per fraction; however, the patient ultimately received only 25 treatments for personal reasons. This case report evaluates the technical workflow of using FBCT-gART for DSRCT and discusses its dosimetric advantages over non-adaptive radiotherapy.

## Introduction

Desmoplastic small round cell tumor (DSRCT) is a rare, highly malignant soft tissue tumor primarily affecting the abdominal and pelvic regions in adolescent males, with a poor prognosis and a high propensity for distant metastasis [1]. The current standard of care involves aggressive multimodal therapies, including surgery, chemotherapy, radiotherapy, intraperitoneal heated chemotherapy, and molecular targeted therapies [2].

Research has shown that whole abdominopelvic radiotherapy (WART) using conventional two-dimensional radiotherapy significantly improves local control and overall survival in patients with locally confined abdominal or pelvic DSRCT following maximal cytoreductive surgery. However, this approach is associated with notable adverse effects, including gastrointestinal and hematological toxicities [3]. Intensity-modulated radiation therapy (IMRT) offers a promising alternative, potentially mitigating these toxicities compared to two-dimensional approaches [2,4]. Despite these advances, only a subset of patients (34%-46%) currently receive radiotherapy [5].

Emerging advanced radiotherapy techniques, such as online adaptive radiotherapy (ART), allow the real-time adjustment of treatment plans based on changes in target volume (TV) and organs-at-risk (OARs) deformations, thereby ensuring optimal target coverage and compliance with OARs dose constraints [6]. This approach is particularly suitable for stereotactic radiotherapy of abdominal and pelvic tumors [7]. Recently, the uRT-linac 506c system [8], which combines kilovoltage fan beam computed tomography (KV-FBCT), a 6 MV X-ray linear accelerator, and a treatment planning system (uRT-TPOIS, R001), has been introduced to enable daily ART. The application of fan beam computed tomography-guided adaptive radiotherapy (FBCT-gART) for DSRCT has not been previously described.

Here, we present the first report on the use of FBCT-gART for DSRCT treatment, detailing the tumor motion dynamics, and discussing the dosimetric advantages over conventional non-adaptive radiotherapy (nART).

## Case presentation

Baseline information

In December 2020, a 33-year-old male presented with abdominal pain of unknown origin. A computed tomography (CT) scan of the chest/abdomen/pelvis revealed a left lower abdominal mass. Laparoscopic exploration and tumor resection revealed a 6 × 4.5 × 3 cm lesion, diagnosed as carcinosarcoma, with a Ki67 staining of 60% and negative for SS18 gene fusion and PDGFRA gene mutation. The patient underwent six cycles of adjuvant therapy with liposomal paclitaxel (240 mg/m²) and epirubicin (130 mg/m²) every three weeks.

The patient presented with abdominal pain five months prior and was referred to oncology. Chest, abdomen, and pelvic CT revealed pelvic masses suggestive of tumor recurrence. Positron emission tomography/computed tomography revealed fluorodeoxyglucose (FDG)-avid lesions below the left diaphragm, left paracolic gutter, below the liver margin, and at the site of a previous abdominal wall hernia. A CT-guided biopsy of the abdominal mass confirmed the diagnosis of a desmoplastic small round cell tumor. Molecular analysis (MSS, EWSR1-WT1 fusion mutation) further supported this diagnosis, staged as stage IV according to the MD Anderson Cancer Center system [9]. After multidisciplinary consultation, the patient underwent combination chemotherapy and targeted therapy, including liposomal doxorubicin (20 mg/m² IV every three weeks), ifosfamide (1.2 g/m² IV daily), and oral anlotinib (12 mg daily). Eleven cycles of treatment were completed. Based on the treatment response and patient preference, a multidisciplinary meeting recommended referral to radiation oncology for consideration of definitive radiotherapy. The prescribed dose regimen was 54 Gy in 27 fractions, delivered at 2 Gy per fraction.

Due to the considerable mobility of abdominal tumors and the risk to adjacent critical OARs, daily online FBCT-gART was selected using the uRT-linac 506c system, which integrates KV-FBCT and a 6 MV X-ray linear accelerator on a unified platform, enabling precise daily adjustments to optimize treatment delivery. The system's capabilities include image fusion, automatic contouring, and planning tools suitable for IMRT and stereotactic body radiation therapy (SBRT), ensuring accurate target coverage and minimal OARs exposure during dose delivery.

Plan preparation and equipment

The patient underwent both MRI and CT dual simulation before treatment, with the CT performed using four-dimensional CT (4D-CT) combined with abdominal compression techniques. He was immobilized with an abdominal pelvic device without contrast. A helical scan protocol covered the abdomen and pelvis with a slice thickness of 3 mm. Treatment planning used volume-modulated arc therapy (VMAT) with two 240° arcs (gantry angles: clockwise from 240° to 120° and counterclockwise from 120° to 240°; collimator angle: 0°; maximum dose rate 600 MU/min) without compensator. Ninety-seven percent (97%) of the planning target volume (PTV) received 100% of the prescription dose (54 Gy in 27 fractions over 6 weeks).

Initial radiotherapy plan formulation

The gross tumor volume (GTV) was defined as visible lesions on the CT simulation image with a uniform volumetric expansion of 0.3 cm to form a PTV. Regarding the contouring of OARs, it started from 1 cm above the topmost lesion and extended to 1 cm below the bottom-most lesion. Relevant OARs included the small bowel, stomach, spinal cord, left kidney, and right kidney. In accordance with standard adaptive radiotherapy practice [10-12], priority was not given to ensuring coverage of the overlapping PTV and OARs. Instead, the PTV was adjusted to avoid overlapping with critical OARs and their surrounding 1 mm margin, particularly sensitive organs, such as the small bowel and stomach, due to their susceptibility to radiation dose distribution.

Online ART plan and simulated non-ART plan formulation

The process began with initial localization and correction using KV-FBCT image guidance to position the patient based on a predefined scan template. The acquired images were then registered with the baseline planned images to correct any localization errors. The online adaptive radiotherapy workflow was then initiated in the treatment planning system (TPS). Within this workflow, the OARs from the baseline plan were automatically contoured by deformation while the target volumes (GTV and PTV) were rigidly mapped onto the current images and manually modified based on the target delineation principles of the baseline plan. Once the regions of interest (ROIs) were confirmed, an adaptive optimization calculation was performed based on the adjusted target areas to generate the current adaptive radiotherapy plan (naming convention: ART PlanX, where X is fractions 1-27). This adaptive optimization algorithm used the dose distribution and clinical goal sheet from the baseline treatment plan as inputs; the clinical goal sheet contained a prioritized wish list specifying the desired prescription dose coverage for the target and the dosimetric constraints for the OARs [8]. When the dose parameters for the ROIs met the predefined goals, the evaluated and optimized plan was transferred to the treatment delivery application for implementation.

**Figure 1:**
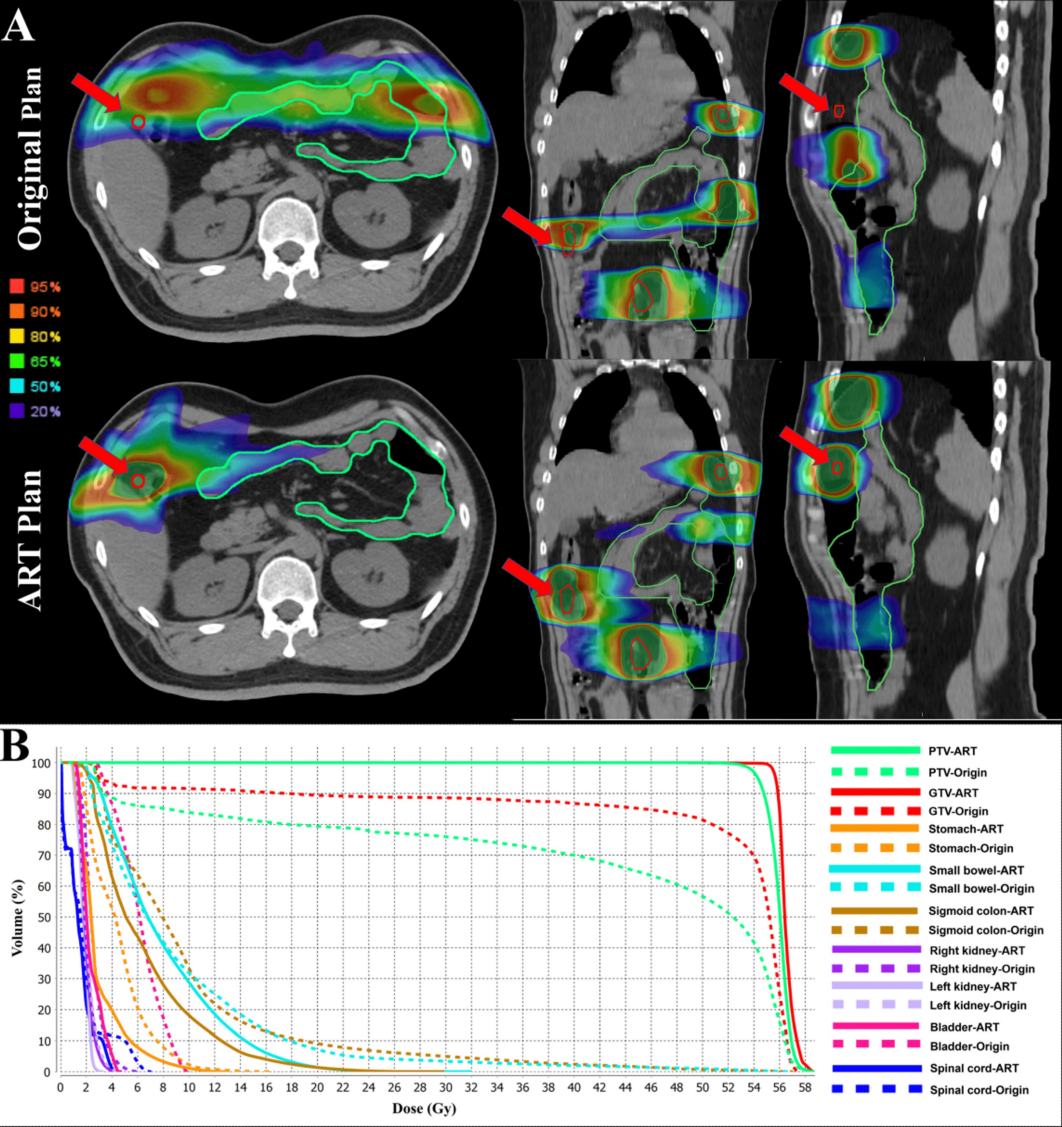
The superiority of FBCT-gART over nART The displayed images are acquired from the initial fraction of radiotherapy, preceding the first daily FBCT scan, and show axial, coronal, and sagittal views (A), the red line outlines the GTV, and red arrows mark the areas of GTV coverage by the ART plans and GTV misses by the nART plans. The DVH curves (B) provide a quantitative comparison between the ART and nART plans, as shown by the solid and dotted lines, respectively. *Abbreviations:* ART: Adaptive radiotherapy; nART: Non-adaptive radiotherapy; FBCT: Fan beam computed tomography; GTV: Gross tumor volume; PTV: Planning target volume; DVH: Accumulated dose-volume histogram

Additionally, a direct calculation was performed by mapping the baseline treatment plan onto the current images to create a simulated nART planX (X=1-27). Figure 1 illustrates the superiority of FBCT-gART over nART at the first radiotherapy fraction. Prior to ART, there is a risk of target area deviation and potentially high radiation exposure to OARs such as the small bowel. After ART, these issues are mitigated, as shown in the dose-volume histogram comparing the ART plan with the nART plan.

Statistical comparison

We conducted a comparative analysis of dose parameters, including GTV, PTV, and OARs, between ART and nART plans, as summarized in Table 1 and Table 2. Each GTV, acquired from daily online contours, was transformed into the planning CT using rigid registration. We then computed the dice similarity coefficient (DSC), Hausdorff distance (HD), and mean distance to agreement (MDA) between the transformed GTV and the baseline GTV in the planning CT.

**Table 1:** Dose parameters comparison of OARs *Abbreviations:* OARs: Organs at risk; nART: Non-adaptive radiotherapy; ART: Adaptive radiotherapy; Dmax: Maximum dose; Deman: Mean dose; V20, V50: The relative volume received equals or exceeds 20Gy, 50Gy

Organs at risk	Dose constraint	nART mean (std dev)	nART median (range)	ART mean (std dev)	ART median (range)	*P* value
Stomach	Dmax < 50 Gy	44.9 (9.4)	48.2 (39.9 - 52.0)	45.3 (9.9)	46.7 (41.2 - 52.8)	0.5780
Small bowel	Dmax < 55 Gy	57.8 (1.2)	57.8 (56.9 - 58.9)	56.1 (0.8)	56.0 (55.4 - 56.7)	<0.0001
Spinal cord	Dmax < 45 Gy	3.7 (1.1)	3.8 (2.7 - 4.1)	3.3 (0.8)	3.3 (2.6 - 3.9)	0.0187
Left kidney	V20 < 20%	0	0	0	0	/
	Dmean < 8Gy	2.5 (0.3)	2.5 (2.2 - 2.8)	2.4 (0.3)	2.5 (2.2 - 2.7)	0.8742
Right kidney	V20 < 20%	0	0	0	0	/
	Dmean < 8Gy	2.4 (0.4)	2.4 (2.1 - 2.7)	2.4 (0.4)	2.5 (2.1 - 2.7)	0.8041
Colon	Dmax < 55 Gy	54.1 (9.4)	57.3 (56.4 - 57.8)	56.7 (2.9)	57.3 (57.0 - 57.5)	0.2011
	V50 < 10%	0.8 (0.5)	0.7 (0.5 - 1.0)	0.9 (0.4)	0.9 (0.7 - 1.0)	0.3702

**Table 2:** Dose parameters comparison of PTV and GTV *Abbreviations: *PTV: Planning target volume; GTV: Gross tumor volume; ART: Adaptive radiotherapy; nART: Non-adaptive radiotherapy; Dmax: Maximum dose; D98, D95, D50, D2: The dose received by 98%, 95%, 50%, 2% of PTV or GTV; V54: The relative volume received equals or exceeds 54Gy. CI: Conformity index; HI: Homogeneity index

Parameters	PTV or GTV	nART mean (std dev)	nART median (range)	ART mean (std dev)	ART median (range)	P value
Dmax	PTV	58.4 (0.9)	58.1 (57.8 - 58.6)	58.1 (0.4)	58.0 (57.8 - 58.4)	0.0894
	GTV	58.1 (0.4)	57.9 (57.6 - 58.4)	57.8 (0.4)	57.7 (57.5 - 58.0)	0.0511
V54(%)	PTV	63.7 (11.2)	64.4 (56.7 - 70.3)	95.5 (0.8)	95.0 (96.0 - 58.0)	<0.0001
	GTV	81.5 (10.2)	82.3 (76.3 - 90.0)	100 (0.04)	100 (100 - 100)	<0.0001
D98	PTV	10.5 (9.0)	6.6 (4.0 - 13.0)	53.6 (0.4)	53.6 (53.4 - 53.7)	<0.0001
	GTV	19.3 (16.2)	13.2 (7.8 - 29.3)	54.8 (0.5)	54.8 (54.6 - 55.1)	<0.0001
D95	PTV	16.7 (13.9)	10.3 (6.5 - 30.0)	54.1 (0.1)	54.0 (54.0 - 54.9)	<0.0001
	GTV	30.3 (17.4)	32.8 (13.8 - 45.1)	55.0 (0.4)	55.0 (54.8 - 55.3)	<0.0001
D50	PTV	54.8 (1.4)	55.0 (54.5 - 55.4)	55.7 (0.3)	55.7 (55.5 - 56.0)	0.0043
	GTV	55.6 (0.7)	55.6 (55.2 - 55.9)	56.0 (0.5)	56.0 (55.7 - 56.3)	0.0069
D2	PTV	57.4 (0.5)	57.4 (57.0 - 57.7)	57.2 (0.2)	57.2 (57.1 - 57.4)	0.1431
	GTV	57.4 (0.5)	57.3 (57.0 - 57.8)	57.3 (0.3)	57.2 (57.2 - 57.5)	0.4216
CI	PTV	0.35 (0.09)	0.37 (0.30 - 0.41)	0.75 (0.03)	0.75 (0.72 - 0.77)	<0.0001
HI	PTV	0.86 (0.17)	0.94 (0.80 - 0.97)	0.06 (0.01)	0.06 (0.06 - 0.07)	<0.0001

In Formula 1, DSC is defined as: \operatorname{DSC}(A, B)=\frac{2|A \cap B|}{|A|+|B|}, where A and B represent the volumes of the baseline and the transformed GTV, respectively.

The DSC quantifies the similarity of the regions of interest (ROIs) on a scale from 0 to 1, with 1 indicating complete overlap between the baseline and transformed GTV.

In Formula 2: h(A, B)=\operatorname{mean}_{a \in A}^{\min }\left\{b_{b \in B}\{d(a, b)\}\right\}, d(a, b) represents the three-dimensional HD between points a and b.

Formula 3: HD(A, B)=\max \{(A, B), h(B, A)\} and

Formula 4: MDA(A, B)=\frac{h(A, B)+h(B, A)}{2}, indicate that both HD and MDA range from 0 to +∞, with values closer to 0, signifying minimal maximum edge displacement of the transformed GTV.

We also assessed the conformity index (CI): CI=\frac{V t, r e f}{V_{t}} \times \frac{V t, r e f}{V_{r e f}}, which measures the agreement between the prescribed dose distribution volume and the target volume, and the homogeneity index (HI) within the target volume: HI=\frac{D_{2}-D_{98}}{D_{50}}.

The CI formula comprises two parts: the first evaluates target volume coverage, and the second assesses the volume of healthy tissue receiving the prescribed dose or higher. The CI ranges from 0 to 1, where a CI of 1 indicates precise target coverage without exceeding the prescribed dose in healthy tissues, and a CI of 0 indicates poor conformity. The HI is used to analyze and quantify the dose homogeneity within the target volume. D2% represents the dose received by the hottest 2% volume of the target area. D98% represents the dose received by the lowest 98% volume of the target area. D50% represents the dose received by the 50% volume of the target area. A value closer to 1 indicates a more uniform dose distribution. A higher HI value may suggest that some areas receive doses that are either too high or too low, indicating greater dose inhomogeneity.

Statistical methods

Statistical analysis using SPSS 25.0 was used to evaluate the dosimetric parameters of GTV, PTV, and OARs. The normality of the data was confirmed using the Shapiro-Wilk test. For normally distributed data, a paired sample t-test was used; for non-normally distributed data, the Wilcoxon signed-rank test was used, with significance set at 0.05.

Results

Movement of the GTV

**Figure 2:**
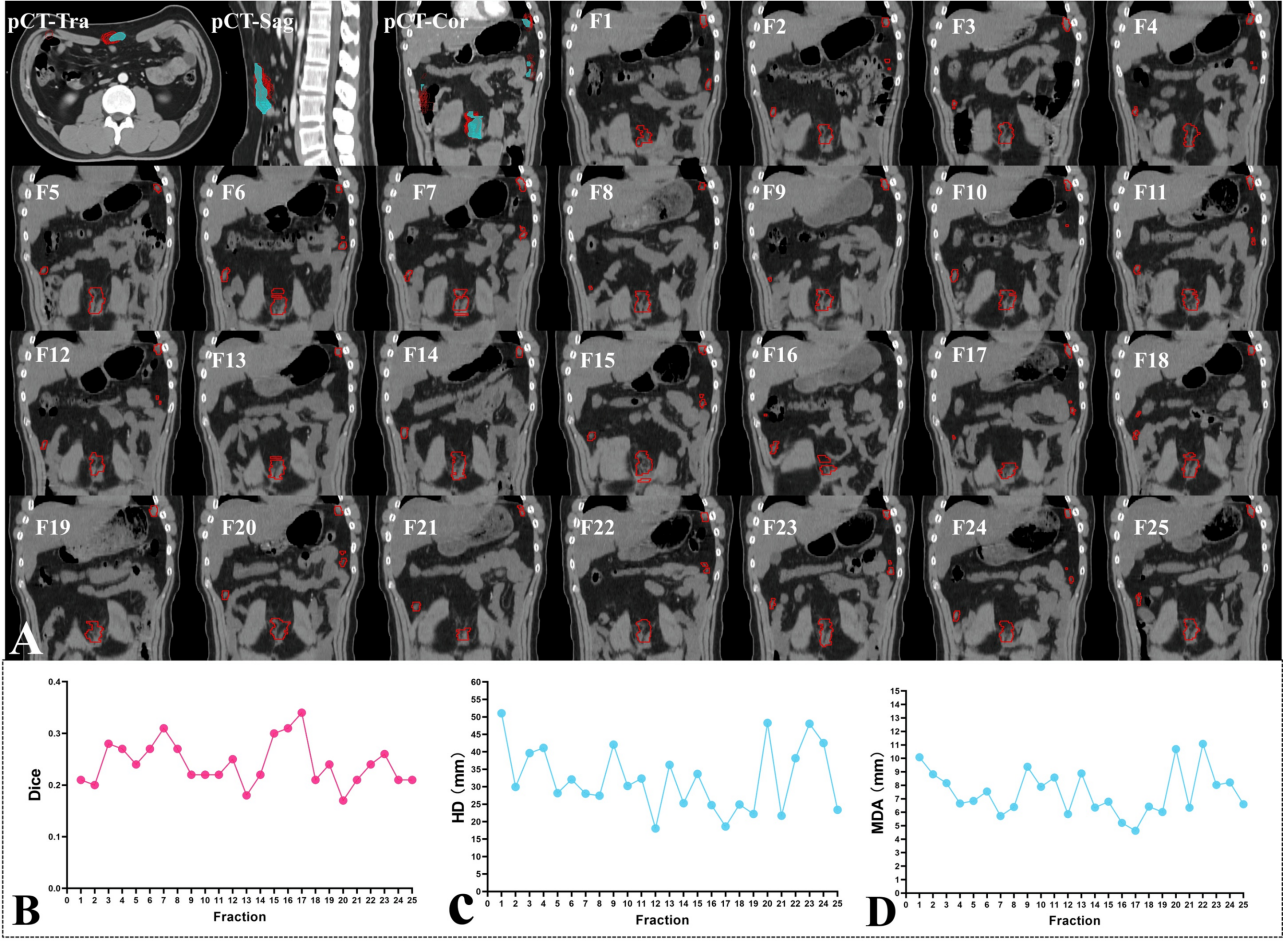
Changes in the position and shape of the GTV during the 25 ART fractions and the corresponding variations in DSC, HD, and MDA In Figure 2A's pCT-Tra, pCT-Sag, and pCT-Cor images, the blue region represents the original GTV while the red line represents all the re-delineated GTVs delineated from daily FBCT (F1-F25) acquired over 25 ART fractions. Figures 2B-2D show the Dice, HD, and MDA variations of the GTV in 25 fractions (compared to the GTV in the planning CT). *Abbreviations: *GTV: Gross tumor volume; ART: Adaptive radiotherapy; DSC: Dice similarity coefficient; HD: Hausdorff distance; MDA: Mean distance to agreement. pCT: Planning computed tomography; Tra: Transverse view; Sag: Sagittal view; Cor: Coronal view; F: Fraction; FBCT: Fan beam computed tomography

**Figure 3:**
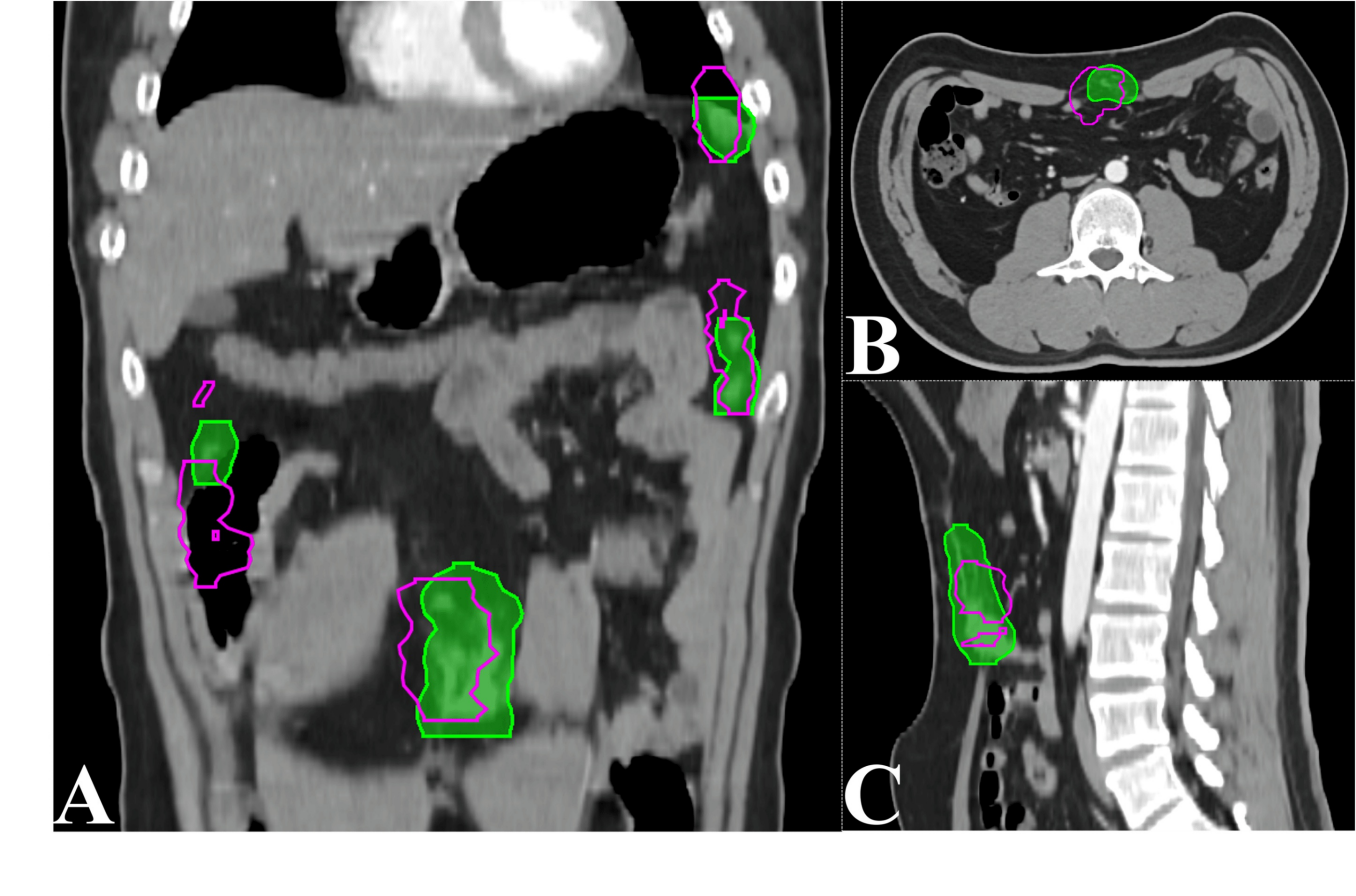
The coverage of the planning CT's PTV for the GTV over 25 fractions A, B, C are planning CT images in coronal, transverse, and sagittal views. The purple lines represent re-delineated GTV delineations from 25 fractions of ART, and the green region represents the PTV from the baseline treatment plan. *Abbreviations:* CT: Computed tomography; PTV: Planning target volume; GTV: Gross tumor volume; ART: Adaptive radiotherapy.

Figure 2A illustrates four lesions in the patient's abdomen showing considerable changes in GTV shape and position over 25 radiotherapy sessions. A more detailed demonstration of the dynamic changes is shown in Video 1. Figures 2B-2D show that during these sessions, the similarity of the GTVs to the baseline plan GTV was significantly low, with a maximum Dice index of 0.34, a mean of 0.24, and a standard deviation of 0.04. The MDA indices indicated considerable variation in GTV edge displacement, with a mean of 7.5 mm and a standard deviation of 1.7 mm. HD showed significant displacement of individual lesions during treatment, with a maximum of 51.0 mm (observed during the first session), an average of 32.3 mm, and a standard deviation of 9.5 mm. Furthermore, Figure 3 shows that the PTV delineated on the planning CT did not adequately cover the changing shapes and positions of the GTV during each treatment fraction. Integrating the GTV delineations from 25 sessions into GTV(all) using Boolean operations resulted in a dice index of only 0.44 compared to the PTV of the planning CT.

**Video 1:** Dynamics of GTV in the uniform coronal view over 25 radiotherapy fractions Red circles: The re-delineated gross tumor volume on each radiotherapy fraction. *Abbreviations:* GTV: Gross tumor volume; F: Fraction

Statistical Results of Dosimetric Parameters

**Figure 4:**
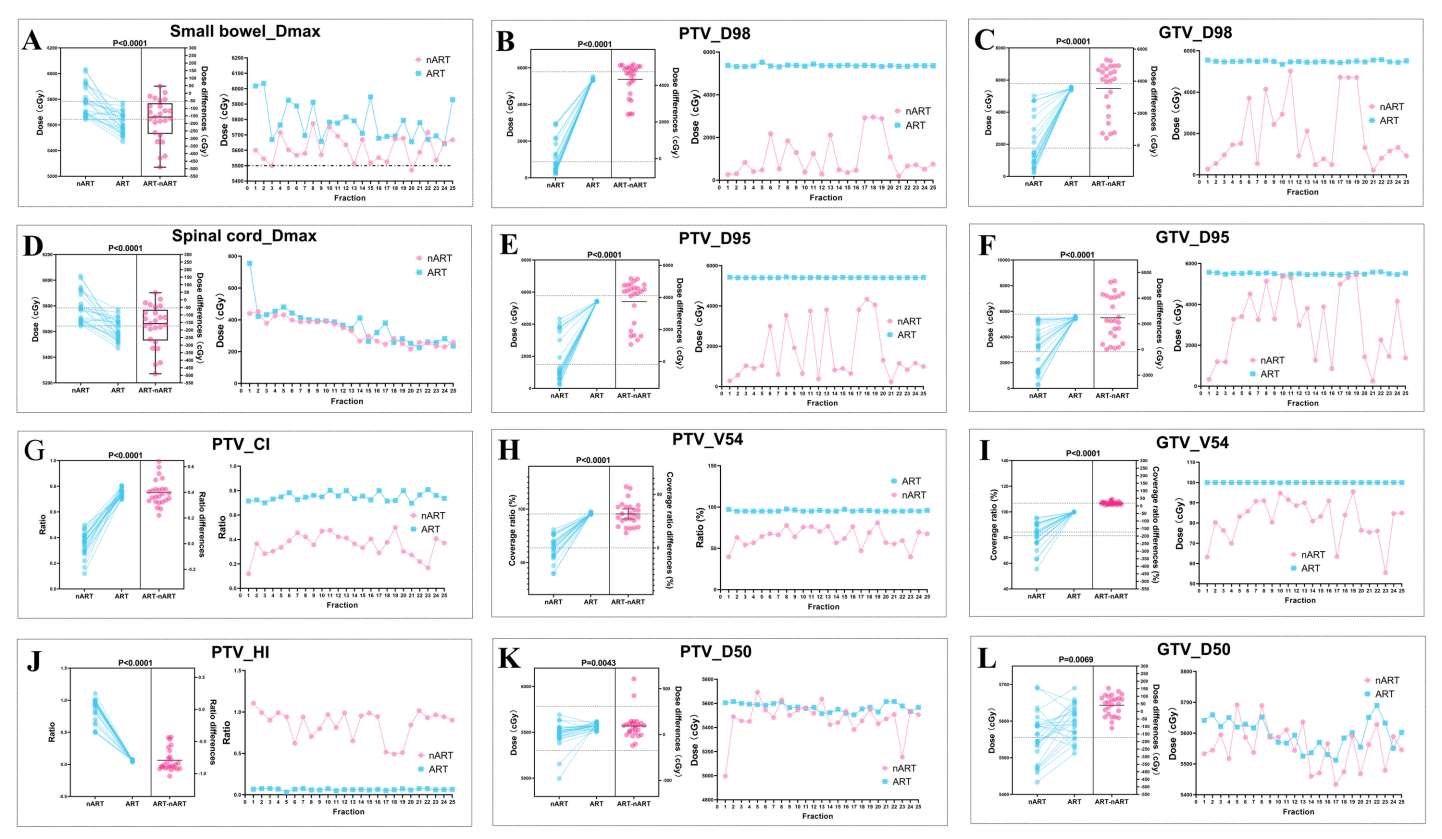
Dosimetric parameters statistically different between ART and nART These estimation plots, a graphical representation of the results of matched samples t-test between ART and nART, contain the raw data as well as a summary of the results of the analysis. A: Dmax of small bowel between ART and nART; B and C: D98 of PTV and GTV between ART and nART, respectively; D: Dmax of the spinal cord between ART and nART; E and F: D95 of PTV and GTV between ART and nART, respectively; G: CI of PTV between ART and nART; H and I: V54 of PTV and GTV between ART and nART, respectively; J: HI of PTV; K and L: D50 of PTV and GTV between ART and nART, respectively. *Abbreviations:* PTV: Planning target volume; GTV: Gross tumor volume; ART: Adaptive radiotherapy; nART: Non-adaptive radiotherapy; Dmax: Maximum dose; D98, D95, D50: The dose received by 98%, 95%, 50% of PTV or GTV; V54: The relative volume received equals or exceeds 54Gy; CI: Conformity Index. HI: Homogeneity index

In terms of dose distribution within the target area, including OARs, PTV, and GTV, as shown in Table 1, Table 2, and Figure 4. ART plans demonstrated superior V54, D98, D95, D50, HI, and CI as compared to nART plans. In terms of OARs sparing, ART plans showed statistically significant advantages over nART plans in terms of Dmax for the small bowel and spinal cord (p < 0.05). In particular, Dmax for the small bowel exceeded the OARs limits in the 25 ART sessions due to partial tumor involvement of the small bowel, regardless of whether median or mean values were used.

Treatment Component Times

**Figure 5:**
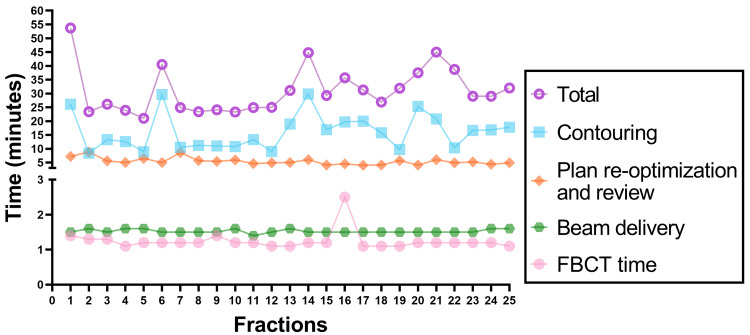
Treatment component times *Abbreviations:* FBCT: Fan beam computed tomography

Treatment component times include FBCT time, contouring time, plan re-optimization and review time, beam delivery time, and total time. These findings are depicted in Figure 5. The mean total treatment time was 31 minutes (standard deviation, 8.3), with the first session being the longest at 53.7 minutes. The subsequent 24 sessions varied mainly due to contouring time.

## Discussion

Radiation therapy for intraluminal tumors is challenging due to inter- and intra-fraction motion caused by patient positioning, bowel and respiratory movements, and variations in bladder filling, leading to uncertainties in the delivered dose [13]. In addition, treatment of intraluminal tumors is limited by the radiation tolerance of adjacent critical organs such as the duodenum, small intestine, and liver [14]. It is therefore essential to recontour and optimize treatment plans on a daily basis, based on the shape and position of both the tumor and critical organs, to achieve safe and effective treatment of abdominal tumors.

While data have been published on other abdominal tumor types treated with MR-guided or cone beam CT (CBCT)-guided adaptive techniques [8,15], clinical reports specific to DSRCT radiotherapy, particularly adaptive therapy cases, remain scarce. This study focuses on the benefits of a novel CT-guided online ART system for DSRCT patients with the aim of reducing toxicity and improving target coverage and dose uniformity.

Despite advances in IMRT that have reduced gastrointestinal toxicity in DSRCT patients, serious side effects can still occur. A retrospective study found that 84% of patients who underwent cytoreductive surgery with heated intraperitoneal chemotherapy followed by IMRT or volumetric modulated arc therapy experienced grade 3 or higher toxicity [16]. In this study, the patient received a prescribed dose of 50 Gy delivered in 25 fractions, five times per week. Real-time image-guided recontouring of the TV and OARs based on image feedback was performed to optimize the dose and deliver treatment efficiently with minimal patient movement. Simulation of nART procedures revealed significant variations in target shape and position compared to planning CT and previous treatments, highlighting the inadequacy of traditional non-adaptive approaches in ensuring adequate target coverage at prescribed doses. In contrast, FBCT-gART improved both target coverage and dose uniformity. The treatment was also well-tolerated, with no significant gastrointestinal toxicity observed within three months after radiotherapy.

The core components of online ART are high quality imaging and precise deformation alignment, facilitated by advanced device functionality and integrated TPS for quick plan turnaround significantly. In this study, the uRT-Linac 506c system integrates diagnostic-quality KV-FBCT with a 6 MV X-ray accelerator, enabling high-quality image guidance and radiation delivery, all of which are supported by integrated ART software. After generating image sequences using FBCT, the corresponding CT values could be directly applied to electron density conversion curves, allowing precise electron density calculations essential for treatment planning [8]. This capability is critical for online ART and cannot be achieved with other imaging modalities, such as CBCT or MRI, but certain CBCT systems (not all) can calculate directly on the CBCT with accurate electron densities. In addition, the coaxial design of the CT and linear accelerator, which share the same table and coordinate system, combined with immersion monitoring and automatic correction, ensures spatial stability throughout the treatment process [8]. The use of the Demons algorithm for deformation image alignment ensures high image quality and precise alignment [17-19].

In this study, FBCT-gART demonstrated significant dosimetric advantages, potentially allowing more precise delivery of higher biologically effective doses to DSRCT patients while limiting gastrointestinal toxicity. This treatment approach aims to expand the use of radiotherapy in DSRCT patients and has the potential to increase the opportunities for combination with other treatment modalities, such as chemotherapy and targeted therapy. However, as this report is a case study with a short follow-up period, we can only confirm the dosimetric advantages of FBCT-gART and cannot yet determine whether these dosimetric advantages translate into clinical benefit. Therefore, further studies with larger sample sizes are needed to validate these findings.

The conditions and workflow for online ART need to be further optimized. With the rapid development of artificial intelligence and deep learning technologies in the field of radiotherapy, it is becoming increasingly possible to automatically and rapidly delineate organs at risk and target areas in the abdomen. This advancement will greatly contribute to the development and efficiency of ART. Automated contouring of abdominal organs at risk and targets will increase precision and consistency, potentially improving treatment outcomes and reducing the burden on clinicians.

## Conclusions

FBCT-gART is an ideal technology for personalized precision radiotherapy, particularly for the ART treatment modality in DSRCT. It demonstrates significant dosimetric advantages, enhancing the role of radiotherapy within comprehensive treatment strategies for DSRCT patients, and has potential for clinical uptake. However, larger clinical trials are needed to fully evaluate its impact on overall survival, progression-free survival, and toxicity in DSRCT patients.
